# High Dimensionality Reduction and Immune Phenotyping of Natural Killer and Invariant Natural Killer Cells in Latent Tuberculosis-Diabetes Comorbidity

**DOI:** 10.1155/2022/2422790

**Published:** 2022-02-21

**Authors:** Gokul Raj Kathamuthu, Nathella Pavan Kumar, Kadar Moideen, Pradeep A. Menon, Subash Babu

**Affiliations:** ^1^National Institutes of Health-NIRT-International Center for Excellence in Research, Chennai, India; ^2^National Institute for Research in Tuberculosis (NIRT), Chennai, India; ^3^Laboratory of Parasitic Diseases, National Institute of Allergy and Infectious Diseases, National Institutes of Health, Bethesda, Maryland, USA

## Abstract

Natural killer (NK) and invariant NKT (iNKT) cells are unique innate lymphocytes that coordinate diverse immune responses and display antimycobacterial potential. However, the role of NK and iNKT cells expressing cytokines, cytotoxic, and immune markers in latent tuberculosis (LTB), diabetes mellitus (DM), or preDM (PDM) and nonDM (NDM) comorbidities is not known. Thus, we have studied the unstimulated (UNS), *Mycobacterium tuberculosis* (Mtb [PPD, WCL]), and mitogen (P/I)-stimulated NK and iNKT cells expressing Type 1 (IFN*γ*, TNF*α*, and IL-2), Type 17 (IL-17A, IL-17F, and IL-22) cytokines, cytotoxic (perforin, granzyme B, and granulysin) and immune (GMCSF, PD-1, and CD69) markers in LTB comorbidities by dimensionality reduction and flow cytometry. Our results suggest that LTB DM and PDM individuals express diverse NK and iNKT cell immune clusters compared to LTB NDM individuals. In UNS condition, frequencies of NK and iNKT cells expressing markers are not significantly different. After Mtb antigen stimulation, NK cell expressing [Type 1 (IFN*γ*, TNF*α*, and IL-2), GMCSF in PPD and IFN*γ* in WCL), Type 17 [(IL-17A), PD-1 in PPD), (IL-17A, IL-17F, and IL-22), PD-1 in WCL], and cytotoxic (perforin, granzyme B in PPD, and WCL)] marker frequencies were significantly reduced in LTB DM and/or PDM individuals compared to LTB NDM individuals. Similarly, iNKT cells expressing [Type 1 (IFN*γ*, IL-2), GMCSF in PPD), TNF*α*, GMCSF in WCL), Type 17 (IL-17A), PD-1 in PPD, IL-17F in WCL) cytokines were increased and cytotoxic or immune (perforin, granzyme B, granulysin), CD69 in PPD, perforin and CD69 in WCL] marker frequencies were significantly diminished in LTB DM and/or PDM compared to LTB NDM individuals. Finally, NK and iNKT cell frequencies did not exhibit significant differences upon positive control antigen stimulation between the study population. Therefore, altered NK cell and iNKT cells expressing cytokines, cytotoxic, and immune markers are characteristic features in LTB PDM/DM comorbidities.

## 1. Introduction

Latent tuberculosis (LTB) is described as a subclinical state of infection with *Mycobacterium tuberculosis*. Likewise, active tuberculosis (ATB) is characterized by the existence of clinical manifestation and infects various parts of the body, most commonly the lungs [1]. Similar to TB-HIV coinfection, the re-emerging risk of TB infection is augmented by Type 2 diabetes mellitus (DM) [2, 3]. Thus, it is well-known that DM condition enhances the risk of ATB and LTB reactivation [4]. In India, the prevalence rate of DM is estimated to reach 123.5 million in the year 2040 [5]. Similarly, depending upon the inhabitants and geographical position, 5–10% of the preDM (PDM) population become diabetic each year [6, 7]. The PDM prevalence was shown to be as high as 25% of individuals among active TB [8]. Therefore, both DM and PDM condition is associated with nexus between metabolic disorders and TB disease.

Human host resistance requires efficient T cell (CD4^+^ and CD8^+^) dependent immune responses against *Mycobacterium tuberculosis* (Mtb) disease [9–11]. Before T cell activation, innate immunity acts as the major defense against invading pathogens upon recognition by diverse pathogen recognition receptors (PRRs). One among them is natural killer (NK) cells and they exhibit memory-type immune markers and form a connection between innate and adaptive immune-mediated response [12]. The NK cells also induce cellular cytotoxicity and cytokine signaling, and they are the important facilitators of innate immunity [12]. They also contribute to the granuloma formation and aid in the host resistance through diverse mechanisms either directly by the release of cytotoxic molecules such as perforin and granulysin, indirectly via adaptive immune signaling, and perhaps via “innate-like memory” cells [12–15].

In addition, during the early disease stages, unconventional T cells play a vital role in providing protection against TB disease [16–18]. Among them, invariant natural killer T (iNKT) cells are specialized innate-like unconventional T cells that respond to both self and foreign glycolipids presented by CD1d as cognate antigens [19]. Their receptor consists of V*α*24 and V*β*11 chains and is potentially activated by *α*-galactosylceramide. The iNKT cells are mainly involved in the formation of granuloma and act as a bridge between innate and adaptive immunity owing to their capability to trigger distinct cell (B cell, dendritic cells, monocytes, T, and natural killer cells) types [20]. They can induce cytokine (mainly Th1 cytokines) release promptly upon activation and without the necessity for secondary stimulation [21, 22] and potentially lyse Mtb infected macrophages and mediate bactericidal effect facilitated by granulysin [23]. The iNKT cell responses were identified in the peripheral blood of TB individuals after stimulation with mycobacterial glycolipid antigens [24]. Reduced frequencies of iNKT cells were reported in pulmonary tuberculosis (PTB) disease in comparison with latent TB (LTB) infection and healthy individuals [13, 25, 16].

However, the role of NK and iNKT cell-mediated immunological response in LTB with DM, PDM, and NDM comorbidities is not studied so far. Therefore, herein, our interest is to examine the frequencies of NK and iNKT cells expressing Type 1 (IFN*γ*, TNF*α*, and IL-2), Type 17 (IL-17A, IL-17F, and IL-22) cytokines, cytotoxic markers (perforin [PFN], granulysin [GNLSN], and granzyme [GZE] B), and other immune markers (GMCSF, PD-1, and CD69) using dimensionality reduction [uniform manifold approximation and projection (UMAP)] and immune phenotyping by flow cytometry.

## 2. Methods

### 2.1. Study Population

We have collected 60 samples from LTB DM, PDM, and NDM coinfected (20 samples in each group) groups, and they were divided based on their glycated hemoglobin (HbA1c) status, based on the American Diabetes Association criteria.  Table 1 displays the study demographics of three different groups. Both tuberculin skin test (TST) and QuantiFERON TB-Gold (Qiagen) in tube ELISA were used to define LTB positivity. The TST positive was calculated by an induration at the site of tuberculin inoculation of at least 5 mm size in diameter. The HbA1c and random blood glucose levels of NDM group is <5.7% and <140 mg/dl; whereas, for PDM the levels are >5.7% to <6.4% and 140–199 mg/dl. Finally, the HbA1c and random blood glucose levels for Type 2 DM group is >6.5%, >200 mg/dl. All the three groups were not affected by active tuberculosis, other lung or systemic disease, and they all had normal chest X-rays. All individuals are BCG vaccinated, with absence of HIV infection, and were nonconsuming of steroids. The National Institute of Research in Tuberculosis (NIRT) Internal Ethics Committee (IEC2011013) approved the study. We also received the informed written consent from all study population.

### 2.2. Peripheral Blood Mononuclear Cell (PBMC) Isolation, Thawing, and Stimulation

Ficoll-Paque centrifugation was used to isolate the PBMCs from the peripheral blood samples, and the cell viability and count were determined using the trypan-blue exclusion assay. The PBMCs were stored at −80°C followed by cryopreservation in liquid nitrogen using fetal bovine serum (FBS, Gibco, Thermo Fisher) and dimethyl sulfoxide (Sigma Aldrich). The PBMC thawing and in vitro antigen (PPD [10 *μ*g/mL], Staten's Serum Institute, WCL [1 *μ*g/mL], BEI Resources) stimulation were performed as previously described [26].

### 2.3. PBMC Staining and Flow Cytometry

The cryopreserved PBMCs were thawed and washed with PBS buffer and later with PBS (Lonza)/1% BSA (Sigma Aldrich). Next, the cells were surface stained and incubated (30–60 min) at 4°C in the dark and washed with permeabilization/wash buffer (BD Biosciences). Later, the cells were stained with intracellular antibodies and incubated for 2 hours at 4°C. The surface and intracellular antibodies were obtained either from BD PharMingen, Invitrogen (eBiosceinces), Miltenyi Biotec, and R&D systems: CD3 (SK7, AmCyan), CD4 (RTA-T4, APC-H7), CD8 (PECy7), CD56 (RTA-T4, APC-H7), 6B11/TCRV*α*24 (PerCp-Vio700), TNF*α* (FITC), IFN*γ* (B27, PE), IL-2 (MQ1-17H12, APC), IL-17A (N49-653, FITC), IL-22 (22URPI, PE), IL-17F (O33-782, Alexa fluor 647), Perforin (FITC), granzyme B (GB11, Alexa fluor 647), granulysin (DH2, PE), granulocyte-macrophage colony-stimulating factor [GM-CSF] (BVD2-21C11, PB), CD279 (PD-1, EH12.2H7, pacific blue, [PB]), and CD69 (FN50, PB). We have given the combination/panel of antibodies used in this study as supplementary Table 1. Once the incubation was over, PBMCs were washed using 1x PBS and the cells were subjected to acquisition. The cells were acquired using eight-colour cytometry with FACSDiva Software version 6 (FACSCanto II). Totally 50,000 gated lymphocyte events were recorded, and they were set using forward vs side scatter. The gating strategy for NK and iNKT cells expressing cytokines, cytotoxic, and immune activation markers was calculated by FMO. We represented the data in figures as frequencies (unstimulated) and absolute frequencies (Mtb antigen stimulations).

### 2.4. Statistical Analysis

High dimensionality reduction (UMAP) analysis was performed for NK cells (4000-CD3^−^CD56^+^ lymphocytes were downsampled) and iNKT cells (5000-CD3^+^ lymphocytes were downsampled) using the FlowJo™ plugins (version 10) available in FlowJo 3 (TreeStar Inc., Ashland, OR). A chi-square test was used to measure the significant expression of cytokines, cytotoxic, and immune markers [mean fluorescence intensity (median values) obtained from UMAP analysis] between the study population. The frequencies of Type 1, Type 17 cytokines, cytotoxic, and immune markers expressing NK and iNKT cell subsets were analyzed using FlowJo™ 3 (version 10). The statistical analysis was performed using GraphPad PRISM (version 9.2) (Graph Pad Software, Inc., San Diego, CA). The central tendency was represented using geometric means (GM), and intergroup comparison was calculated using nonparametric Kruskal-Wallis test.

## 3. Results

### 3.1. LTB DM/PDM Individuals Are Characterized by Differential Expression and Diminished ﻿ Frequencies of NK Cells Expressing Type 1 Cytokine and GMCSF Immune Marker

**Figure 1:**
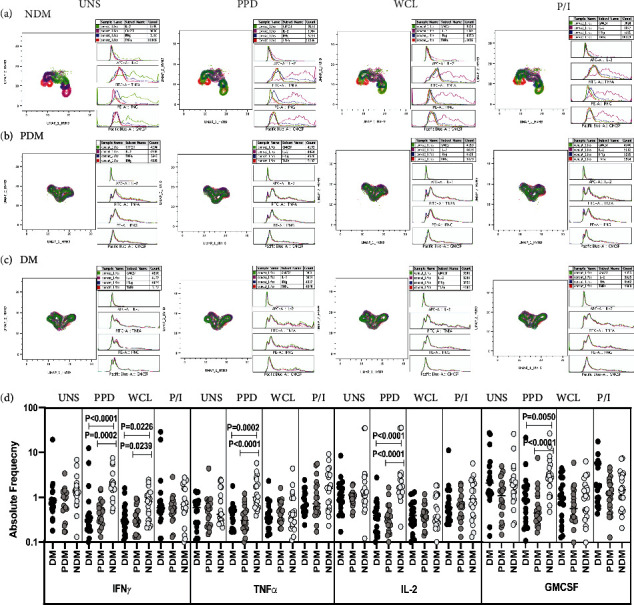
High dimensionality UMAP analysis of NK cells expressing Type 1 cytokine and GMCSF immune marker. (a) NDM, (b) PDM, and (c) DM expression and histogram profile or dot blot expression of NK cells expressing Type 1 cytokine [TNF*α* (red), IFN*γ* (blue), IL-2 (pink)] and GMCSF (green) clusters upon UNS, PPD, WCL antigen, and (mitogen) P/I stimulation. (d) Reduced frequencies of NK cells expressing Type 1 cytokine and GMCSF immune marker in LTB comorbid individuals. Peripheral blood mononuclear cells (PBMCs) was either unstimulated (UNS) or stimulated with *Mycobacterium tuberculosis* (Mtb) [PPD, WCL] antigens or mitogen for 18 h. The UNS and Mtb antigen-stimulated frequencies of Type 1 (IFN*γ*, TNF*α*, and IL-2) cytokines and GMCSF immune marker were examined in LTB DM (*n* = 20), LTB PDM (*n* = 20), and LTB NDM (*n* = 20) individuals. Each black, grey, and silver colour circle indicates a single individual and the bars represent the geometric mean values. Net frequencies were determined by subtracting the UNS from antigen stimulated frequencies for each individual. *P* values were calculated using the Kruskal-Wallis test with Dunn's multiple comparisons.

The representative plots of gated NK cells expressing Type 1, Type 17 cytokines, cytotoxic, and immune markers among LTB (DM, PDM, NDM) comorbid individuals are shown using high dimensionality (two-dimensionally, UMAP 1 vs UMAP 2) UMAP analysis (Supplementary Figure 1(a)). The expression of NK cells expressing Type 1 (IFN*γ*, IL-2, and TNF*α*) cytokines and GMCSF immune marker is significantly different between the study groups (Figures 1(a)–1(c)).

The gating strategy and representative plots of NK cells expressing Type 1 (IFN*γ*, IL-2, and TNF*α*), Type 17 (IL-17A, IL-17F, IL-22) cytokines, cytotoxic (perforin [PFN], granzyme [GZE] B, granulysin [GNLSN]), and immune (GMCSF, PD-1, and CD69) markers are shown (Supplementary Figure 2). We also show the FMO controls of activation (a–d: CD3, CD4, CD8, and CD56) markers in (Supplementary Figure 3). We used multicolour flow cytometry to examine the unstimulated (UNS) and Mtb antigen (PPD, WCL) and mitogen (P/I)-stimulated frequencies of NK cell expressing Type 1 cytokine and GMCSF immune marker in LTB (NDM, PDM and DM) comorbid individuals (Figure 1(d)). There was no significant difference in the frequencies of NK cells expressing Type 1 cytokine and GMCSF immune marker upon unstimulated condition among LTB (NDM, PDM, and DM) comorbid groups. The frequencies of NK cells expressing Type 1 cytokine and GMCSF were significantly diminished in PPD [(IFN*γ*, TNF*α*, and IL-2), (GMCSF)] and WCL (TNF*α*) antigen stimulation in LTB DM and PDM individuals than LTB NDM individuals. There was no significant difference in the frequencies of NK cells expressing Type 1 cytokine and GMCSF upon mitogen stimulation among LTB comorbid group (Figure 1(d)). Therefore, diminished NK cell expressing Type 1 cytokine and GMCSF immune marker frequencies are the characteristic feature of LTB (DM and PDM) groups.

### 3.2. LTB DM/PDM Individuals Are Characterized by Differential Expression and Elevated Frequencies of iNKT Cells Expressing Type 1 Cytokine and GMCSF Immune Marker

**Figure 2:**
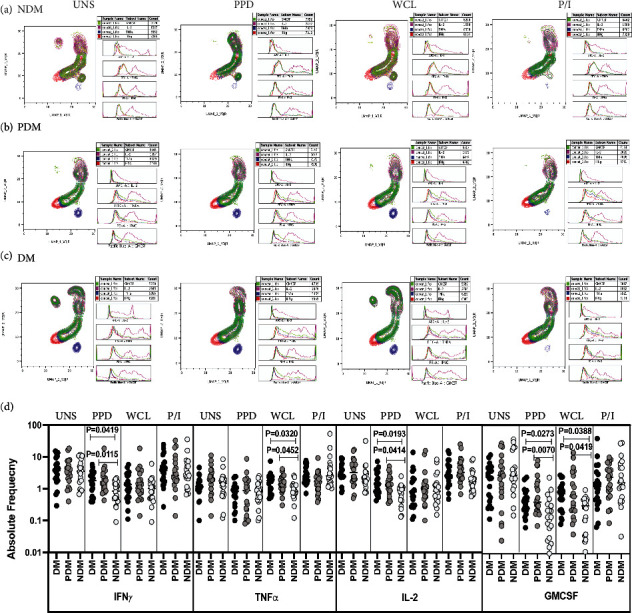
High dimensionality UMAP analysis of iNKT cells expressing Type 1 cytokine and GMCSF immune marker. (a) NDM, (b) PDM, and (c) DM expression and histogram profile or dot blot expression of iNKT cells expressing Type 1 cytokine [TNF*α* (red), IFN*γ* (blue), IL-2 (pink)] and GMCSF (green) clusters upon UNS, PPD, WCL antigen, and mitogen stimulation. (d) Elevated frequencies of iNKT cells expressing Type 1 cytokine and GMCSF immune marker in LTB comorbid individuals. PBMCs were either UNS or stimulated with Mtb [PPD, WCL] antigens and mitogen for 18 h. The UNS and Mtb antigen-stimulated frequencies of Type 1 (IFN*γ*, TNF*α*, and IL-2) cytokines and GMCSF immune marker were examined in LTB DM (*n* = 20), LTB PDM (*n* = 20), and LTB NDM (*n* = 20) individuals. Each black, grey, and silver colour circle indicates each group, and geometric mean was indicated using bars. The frequencies of net cytokine were determined by subtracting the UNS cytokine frequency from Mtb and mitogen antigen stimulated frequencies. The significant difference was measured using the Kruskal-Wallis test Dunn's multiple comparisons, and they were mentioned using *P* values.

The representative plots of gated iNKT cells expressing Type 1, Type 17 cytokines, cytotoxic, and immune markers among LTB (DM, PDM, and NDM) comorbid individuals are shown using high dimensionality UMAP analysis (Supplementary Figure 1A-II). The expression of iNKT cells expressing Type 1 (IFN*γ*, IL-2, and TNF*α*) cytokines and GMCSF immune marker is significantly different between the study groups (Figures 2(a)–2(c)).

We show the iNKT cells expressing Type 1, Type 17 cytokines, cytotoxic, and immune marker gating strategy and representative plots in Supplementary Figure 2. The frequencies of iNKT cells expressing Type 1 cytokine and GMCSF did not significantly differ between the study groups in unstimulated condition (Figure 2(d)). The frequencies of iNKT expressing Type 1 cytokine and GMCSF were significantly elevated in PPD [(IFN*γ* and IL-2), GMCSF] WCL [(TNF*α*), GMCSF] antigen stimulation in LTB (DM, PDM) comorbid group in comparison with LTB NDM individuals. However, after mitogen stimulation, iNKT cells expressing Type 1 cytokine and GMCSF frequencies were not significantly different between LTB comorbid individuals (Figure 2(d)). Thus, frequencies of Type 1 cytokine and GMCSF expressing iNKT cells are increased in LTB DM and PDM individuals.

### 3.3. LTB DM/PDM Individuals Are Characterized by Differential Cluster Expression and Reduced NK Cells Expressing Type 17 Cytokines and PD-1 Immune Marker

**Figure 3:**
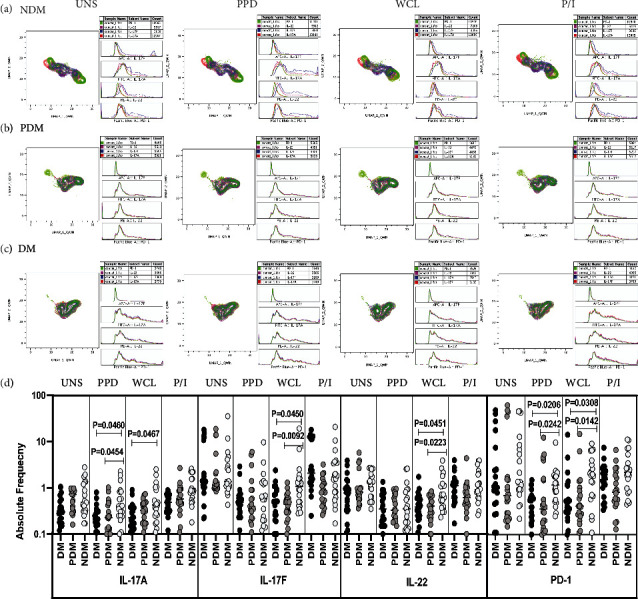
High dimensionality UMAP analysis of NK cells expressing Type 17 cytokines and PD-1 immune marker. (a) NDM, (b) PDM, and (c) DM expression and histogram profile or dot blot expression of NK cells expressing Type 17 cytokines [IL-17A (red), IL-17F (blue), IL-22 (pink)] and PD-1 (green) clusters upon UNS, PPD, WCL, and mitogen antigen stimulation. (d) Reduced frequencies of NK cells expressing Type 17 cytokines and PD-1 immune marker in LTB comorbid individuals. PBMCs were either UNS or stimulated with Mtb [PPD, WCL] or mitogen antigens for 18 h. The UNS and Mtb antigen-stimulated frequencies of Type 1 (IL-17A, IL-17F, and IL-22) cytokines and PD-1 immune were examined in LTB DM (*n* = 20), LTB PDM (*n* = 20), and LTB NDM (*n* = 20) individuals. Each black, grey, and silver colour circle indicates each group, and geometric mean was indicated using bars. The frequencies of net cytokine were determined by subtracting the UNS cytokine frequency from Mtb and mitogen antigen stimulated frequencies. The significant difference was measured using the Kruskal-Wallis test Dunn's multiple comparisons, and they were mentioned using *P* values.

The expression of NK cells expressing Type 17 (IL-17A, IL-22, and IL-17F) cytokines and PD-1 immune marker is significantly different between the study groups (Figures 3(a) and 3(c)).

We analyzed the NK cells expressing Type 17 (IL-17A, IL-17F, and IL-22) cytokines and PD-1 immune marker frequencies in LTB (NDM, PDM, and DM) coinfected individuals in unstimulated, Mtb antigen, and mitogen stimulated conditions (Figure 3(d)). There was no significant difference in UNS condition for NK cells expressing Type 17 cytokines and PD-1 immune marker between LTB coinfected groups. In contrast, LTB DM and PDM individuals exhibited significantly reduced frequencies of NK cells expressing Type 17 cytokines and PD-1 in PPD [(IL-17A), PD-1) and WCL (IL-17A, IL-17F, and IL-22), (PD-1)] antigen stimulation in comparison with LTB NDM group. After mitogen stimulation, there was no significant difference in the NK cells expressing Type 17 cytokines and PD-1 immune marker frequencies between the study groups (Figure 3(d)). Hence, LTB DM and PDM comorbid individuals are associated with diminished Type 17 expressing cytokine and PD-1 immune marker frequencies.

### 3.4. LTB DM/PDM Individuals Are Characterized by Differential Cluster Expression and Increased iNKT Cells Expressing Type 17 Cytokines and PD-1 Immune Marker

**Figure 4:**
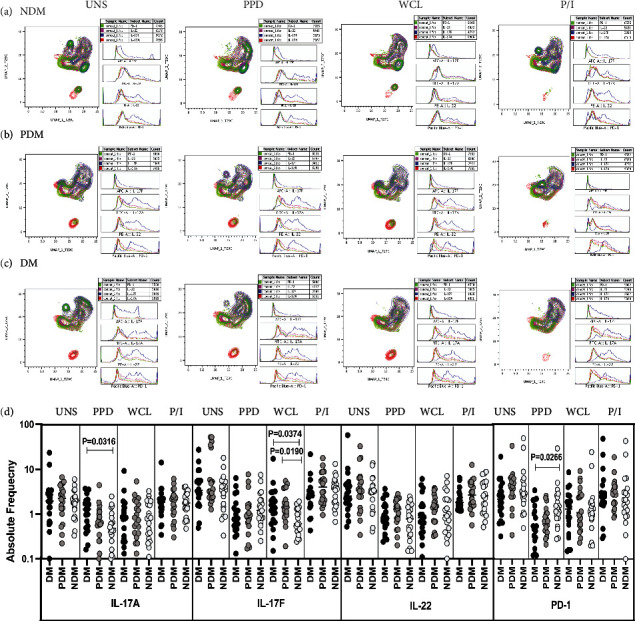
High dimensionality UMAP analysis of iNKT cells expressing Type 17 cytokines and PD-1 immune marker. (a) NDM, (b) PDM, and (c) DM expression and histogram profile or dot blot expression of iNKT cells expressing Type 17 cytokines [IL-17A (red), IL-17F (blue), IL-22 (pink)] and PD-1 (green) clusters upon UNS, PPD, WCL, and mitogen antigen stimulation. (d) Elevated frequencies of iNKT cell expressing Type 17 cytokines and PD-1 immune marker in LTB comorbid individuals. PBMCs were either UNS or stimulated with Mtb [PPD, WCL] or mitogen antigens for 18 h. The UNS and Mtb antigen-stimulated frequencies of Type 17 (IL-17A, IL-17F, and IL-22) cytokines and PD-1 immune were examined in LTB DM (*n* = 20), LTB PDM (*n* = 20) and LTB NDM (*n* = 20) individuals. Each black, grey, and silver colour circle indicates each group, and geometric mean was indicated using bars. The frequencies of net cytokine were determined by subtracting the UNS cytokine frequency from Mtb and mitogen antigen stimulated frequencies. The significant difference was measured using the Kruskal-Wallis test Dunn's multiple comparisons and they were mentioned using P values.

The expression of iNKT cells expressing Type 17 (IL-17A, IL-22, and IL-17F) cytokines and PD-1 immune marker is significantly different between the study groups (Figures 4(a)–4(c)).

We studied the frequencies of iNKT cells expressing Type 17 (IL-17A, IL-17F, and IL-22) cytokines and PD-1 immune marker in LTB coinfected (NDM, PDM, and DM) individuals with baseline (UNS), after stimulation with mycobacterial antigen (PPD and WCL) and mitogen through multicolour flow cytometry (Figure 4(d)). We describe herein that the frequencies of iNKT expressing Type 17 cytokines were found to be nonsignificant in UNS condition between the LTB comorbid groups. The frequencies of iNKT expressing Type 17 cytokines were significantly increased, and PD-1 immune marker was decreased upon PPD [(IL-17A), PD-1] and WCL (IL-17F) antigen-stimulated conditions in LTB comorbid with PDM and DM group compared to LTB NDM group. Finally, in mitogen stimulation, iNKT cells expressing Type 17 cytokines and PD-1 immune marker frequencies did not display a significant difference between LTB (NDM, PDM, and DM) comorbid individuals (Figure 4(d)). Thus, iNKT cell expressing Type 17 cytokines and PD-1 immune marker were significantly altered between LTB comorbid individuals.

### 3.5. LTB DM/PDM Individuals Are Characterized by Differential Expression and Diminished NK Cells Expressing Cytotoxic and CD69 Immune Marker

**Figure 5:**
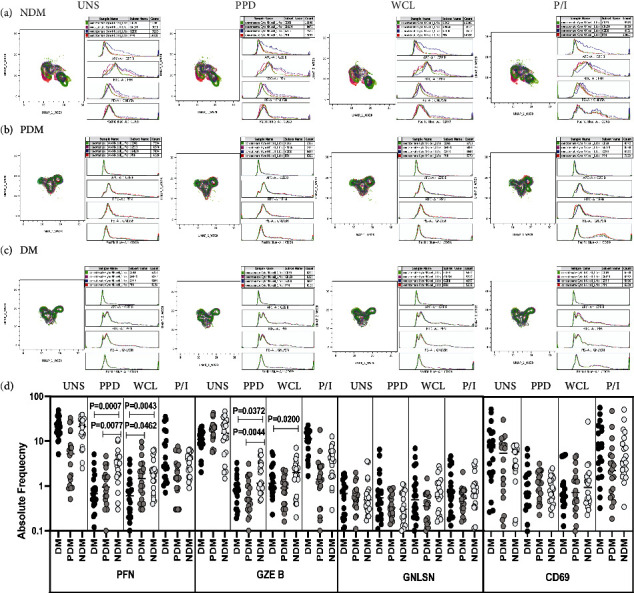
High dimensionality UMAP analysis of NK cells expressing cytotoxic and CD69 immune marker. (a) NDM, (b) PDM, and (c) DM expression and histogram profile or dot blot expression of NK cell expressing cytotoxic [PFN (red), GZE B (blue), GNLSN (pink)] and CD69 (green) marker clusters upon UNS, PPD, WCL, and mitogen antigen stimulation. (d) Reduced frequencies of NK cell expressing cytotoxic and CD69 immune marker in LTB comorbid individuals. The PBMCs were cultured using unstimulated, stimulated with Mtb antigens and mitogen antigens for 18 h, and the cytotoxic (PFN, GZE B, and GNLSN) and CD69 immune markers frequencies were determined. The unstimulated, PPD, WCL and mitogen antigen stimulated conditions in LTB DM (*n* = 20), LTB PDM (*n* = 20), and LTB NDM (*n* = 20) individuals are displayed. Each black, grey, and silver colour circle indicates each group, and geometric mean weas indicated using bars. The frequencies of net cytokine were determined by subtracting the UNS cytokine frequency from Mtb and mitogen antigen stimulated frequencies. The significant difference was measured using the Kruskal-Wallis test Dunn's multiple comparisons and they were mentioned using *P* values.

The expression of NK cells expressing cytotoxic markers (PFN, GZE B, and GNLSN) and CD69 immune marker are significantly different between the study groups (Figures 5(a)–5(c)).

To determine the NK cell expressing cytotoxic markers in LTB (NDM, PDM, and DM) comorbid group, flow cytometry was used to delineate the NK cell expressing cytotoxic marker frequencies upon UNS, stimulated with Mtb antigen-specific antigens (PPD and WCL) and mitogen (Figure 5(d)). There was no significant difference in the frequencies of NK cells expressing cytotoxic and CD69 immune markers in LTB coinfected groups. In contrast, after PPD and WCL (PFN and GZE B) antigen stimulation, the net frequencies of NK cells expressing cytotoxic markers were significantly diminished in LTB DM and/or LTB PDM group in comparison with LTB NDM group. There was no significant difference in NK cell expressing cytotoxic marker frequencies in LTB comorbid individuals after mitogen stimulation (Figure 5(d)). Therefore, LTB DM and PDM individuals are associated with decreased frequencies of NK cells expressing cytotoxic markers.

### 3.6. LTB DM/PDM Individuals Are Characterized by Differential Expression and Diminished iNKT Cells Expressing Cytotoxic and CD69 Immune Marker

**Figure 6:**
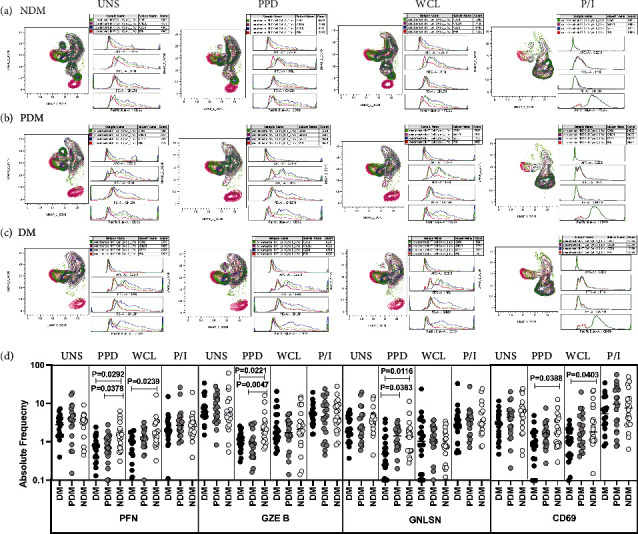
High dimensionality UMAP analysis of iNKT cells expressing cytotoxic and CD69 immune marker. (a) NDM, (b) PDM, and (c) DM expression and histogram profile or dot blot expression of iNKT cells expressing cytotoxic [PFN (red), GZE B (blue), GNLSN (pink)] and CD69 (green) marker clusters upon UNS, PPD, WCL, and mitogen antigen stimulation. (d) Reduced frequencies of iNKT cells expressing cytotoxic and CD69 immune marker in LTB comorbid individuals. The PBMCs were cultured using unstimulated, stimulated with Mtb antigens and mitogen antigens for 18 h, and the cytotoxic (PFN, GZE B, and GNLSN) and CD69 immune markers frequencies were determined. The unstimulated, PPD, WCL, and mitogen antigen stimulated conditions in LTB DM (*n* = 20), LTB PDM (*n* = 20), and LTB NDM (*n* = 20) individuals are displayed. Each black, grey, and silver colour circle indicates each group, and geometric mean was indicated using bars. The frequencies of net cytokine were determined by subtracting the UNS cytokine frequency from Mtb and mitogen antigen stimulated frequencies. The significant difference was measured using the Kruskal-Wallis test Dunn's multiple comparisons, and they were mentioned using *P* values.

The expression of iNKT cells expressing cytotoxic markers (PFN, GZE B, and GNLSN) and CD69 immune markers are significantly different between the study groups (Figures 6(a)–6(c)).

We used multicolour flow cytometry to determine the iNKT cells in LTB comorbid individuals in unstimulated, Mtb antigen (PPD and WCL), and mitogen induced frequencies expressing perforin (PFN), granzyme B (GZE B), and granulysin (Figure 6(d)) markers. There was no significant difference in the frequencies of iNKT cells expressing cytotoxic markers between study individuals in UNS condition (Figure 6(d)). The net frequencies of iNKT cells expressing cytotoxic markers (PFN, GZE B, and GNLYSN) were significantly diminished upon PPD [(PFN, GZE B, and GNLSN), CD69] and WCL [(PFN), CD69] antigen stimulation in LTB DM and PDM groups in comparison with LTB NDM group. The frequencies of iNKT cells expressing cytotoxic and immune markers were not significantly different in LTB DM group compared to LTB PDM and NDM group (Figure 6(d)). Hence, LTB DM and PDM individuals are characterized by diminished frequencies of iNKT cells expressing cytotoxic and CD69 immune markers.

## 4. Discussion

NK cells provide nexus between innate and adaptive immune response against Mtb disease [15, 27, 28]. They also activate IFN*γ* and TNF*α* to induce protective immunity and mediate an efficient killing of target cells by lytic (through perforin and granzymes) mechanisms [29, 30]. They have the ability to kill target molecules by interacting with antigen-presenting T cells; hence, they might be involved in single or multiple steps of immune-mediated attack [31]. Similarly, iNKT cells are very important for host resistance against TB disease and numerous microbial pathogens as well [32, 33]. Previous studies have shown significant (either elevated or diminished) alterations in the iNKT cells in TB disease [25, 34]. Hence, they could potentially contribute to the immunopathology of TB disease by a different mechanism like recognizing foreign or self-lipid antigens mediated via CD1d molecule or upon activation by native cytokine complexes [35]. However, the detailed role of NK and iNKT cells expressing Type 1, Type 17 cytokines, cytotoxic, and immune markers in LTB with NDM, PDM, and DM comorbidities is not known. Therefore, we have studied the same in this current study. UMAP analysis reveals diverse expression profiles of NK and iNKT cells expressing cytokines, cytotoxic, and immune markers in LTB NDM and PDM individuals compared to DM comorbid individuals. Our data also illustrates that NK cell and iNKT cell expressing Type 1, Type 17 cytokines, cytotoxic, and immune marker frequencies were significantly altered in LTB DM and PDM compared to LTB NDM individuals.

Our findings suggest that LTB comorbid individuals have unique Type 1 cytokine and GMCSF immune clusters expressing NK cell and iNKT cells on UMAP analysis. Further, the frequencies of NK (increased) and iNKT (decreased) cells expressing Type 1 cytokine and GMCSF in LTB DM and LTB PDM groups were altered compared to the LTB NDM group. The changes observed in Type 1 cytokine, and GMCSF frequencies are antigen-specific and similar changes were not reported in unstimulated and mitogen stimulation between the LTB comorbid groups. The decreased NK cell marker expressing frequencies might contribute to the increased severity of disease pathogenesis in DM and PDM comorbid patients, and they might compromise the correlates of immune protection in LTB individuals. Elevated frequencies of iNKT expressing Type 1 (TNF*α* and IL-2) cytokines in the DM and PDM comorbid group indicates they might induce the pathogenesis among LTB comorbid individuals. Previously, it was shown that elevated iNKT cell percentages were reported in diabetic TB patients and therefore exhibit strong antimycobacterial potential through IFN*γ* secretion and lysis of infected macrophages and granulysin expression [36, 37]. In a previous study, they have shown that iNKT cell expressing IFN*γ* was significantly higher in active TB disease compared to LTB and healthy controls [38].

Our data also corroborates that NK and iNKT cells expressing Type 17 cytokine frequencies and PD-1 immune marker was significantly altered in LTB DM and PDM group upon PPD and/or WCL antigen stimulation. Previous data reported that J*α*18/J*α*281-knockout (KO) mice with iNKT cell deficiency did not acquire substantial differences in the respiratory pathology and microbial burden upon infection challenge between KO and wild type (WT) mice. Thus, deficit of the iNKT cells did not alter the consequence of Mtb infection, suggesting they present a state of redundancy for an optimal immune response [39, 40]. Nevertheless, this data implies the association of iNKT expressing Type 17 cells in LTB (DM, PDM, and NDM) comorbidity.

Our results exhibit that cytotoxic and PD-1 marker expression was significantly different between the LTB comorbid individuals. Also, LTB DM and PDM comorbid group showed diminished frequencies of NK cell and iNKT cells expressing cytotoxic (PFN, GZEB, and GNLSN) and PD-1 immune marker after stimulation Mtb antigen (PPD and/or WCL) in comparison with LTB NDM group. Also, active TB coinfected with HIV disease showed decreased numbers of iNKT cells in the peripheral blood [13]. To summarize, downregulation of NK cell and iNKT cells expressing cytotoxic and CD69 immune markers was the characteristic feature of LTB DM and PDM individuals and might be associated with compromised protective immunity, higher antigenicity, and increased disease pathogenesis in LTB comorbid individuals. In addition to the above finding, using similar samples, we showed recently that LTB DM and PDM groups were characterized by decreased *γδ* T cell frequencies expressing Type 1/Type 17 cytokines, cytotoxic, and immune markers in comparison with LTB NDM, indicating that the comorbidities suppress the immune response among LTB individuals [26].

Altogether, we show that DM and PDM comorbidities are associated with altered Type 1, Type 17 cytokines, cytotoxic, and immune markers and possibly correlated with poor correlates of immune protection and elevated mycobacterial pathogenesis induced by prediabetes and diabetes in LTB individuals. Our future direction is to study the association of these NK and iNKT cells in active TB disease comorbid with DM, PDM, and NDM comorbidities. In addition, our study also had limitation because we have used nonglycolipid (PPD, WCL, due to our primary study interest) antigens to stimulate the NK and iNKT cell immune responses rather than the glycolipid antigens which are well known molecules to stimulate the iNKT cells. Thus, this could be one possible reason that we were able to find significant differences in some of the cytokines or cytotoxic markers. Therefore, to understand further, we will explore the role of lipid antigens in modulating iNKT cell immune responses in LTB DM/PDM comorbidities in our future studies.

## Figures and Tables

**Table 1:** Demographics of the study population.

Parameters	LTB NDM	LTB PDM	LTB DM
Number of subjects recruited (*n*)	20	20	20
Gender (M/F)	10/10	11/9	11/9
Median age in years (range)	39.6 (24–62)	45.9 (25–62)	47.1 (25–60)
Glycated hemoglobin level (%)	5.48 (>5.0–5.7)	6.1 (>5.7–6.3)	8.43 (>6.50–11.96)
TST (mm)	>5	>5	>5
QuantiFERON-TB gold assay	Positive	Positive	Positive
BMI	24.0 (17.2–30.9)	24.7 (16.5–36.1)	32.5 (21.5–35.0)

## Data Availability

All relevant data are available within the manuscript and supplementary files.

## Supplementary Materials

Supplementary 1 Figure S1: high dimensionality profile of gated NK and iNKT cells expressing Type 1/Type 17 cytokines, cytotoxic and immune markers using UMAP analysis in LTB comorbid individuals. A-C (I) PBMCs (lymphocytes) were gated for single cells (FSC-A vs FSC-H) and from which NK cells (4000-CD3^−^CD56^+^ population]) and iNKT cells (5000-CD3^+^ population) were downsampled. The downsampled population were concatenated and all the analysis was performed using the inbuilt plugins available in FlowJo™ 3 (Version 10). Overall gated UMAP profile of CD56^+^ NK cell population showing the percentage of expression from CD3^−^ lymphocytes and further gated for Type 1 cytokine [(TNF*α*, IFN*γ*, IL-2) and GMCSF], Type 17 cytokines [(IL-17A, IL-17F, IL-22) PD-1] and cytotoxic [(PFN, GZE B, GNLSN) and CD69] markers percentage of immune clusters in LTB NDM, LTB PDM and LTB DM individuals. A-C (II) Overall gated UMAP profile of iNKT (TCRV⍺24^+^) cell population showing the percentage of expression from CD3^+^ lymphocytes and further gated for Type 1 cytokine [(TNF*α*, IFN*γ*, IL-2) and GMCSF], Type 17 cytokines [(IL-17A, IL-17F, IL-22) PD-1] and cytotoxic [(PFN, GZE B, GNLSN) and CD69] markers percentage of immune clusters in LTB NDM, LTB PDM and LTB DM individuals.

Supplementary 2 Figure S2: PBMCs gating strategy and representative plots for NK and iNKT cell expressing Type 1, Type 17 cytokines, cytotoxic and immune markers. Single cells were gated from lymphocytes and further gated on CD3^−^CD56^+^ population for NK cells; whereas CD3^+^ T cell population were selected and further iNKT cells was gated using TCRV*α*24 versus (vs) side scatter (SSC)-A and the frequencies of different cytokines, cytotoxic and other immune markers were indicated.

Supplementary 3 Figure S3: FMO activation (A. CD3, B. CD4, C. CD8 and D. CD56) markers used in the study.

Supplementary 4 Table S1: combination/panel of NK and iNKT cell antibodies used in the study.
