# Supplementary figures and images for: High Dimensionality Reduction and Immune Phenotyping of Natural Killer and Invariant Natural Killer Cells in Latent Tuberculosis-Diabetes Comorbidity
Source: J Immunol Res. 2022 Feb 21;2022:2422790. doi: 10.1155/2022/2422790 (PMC8886750; doi:10.1155/2022/2422790)

# Supplementary Figure 1

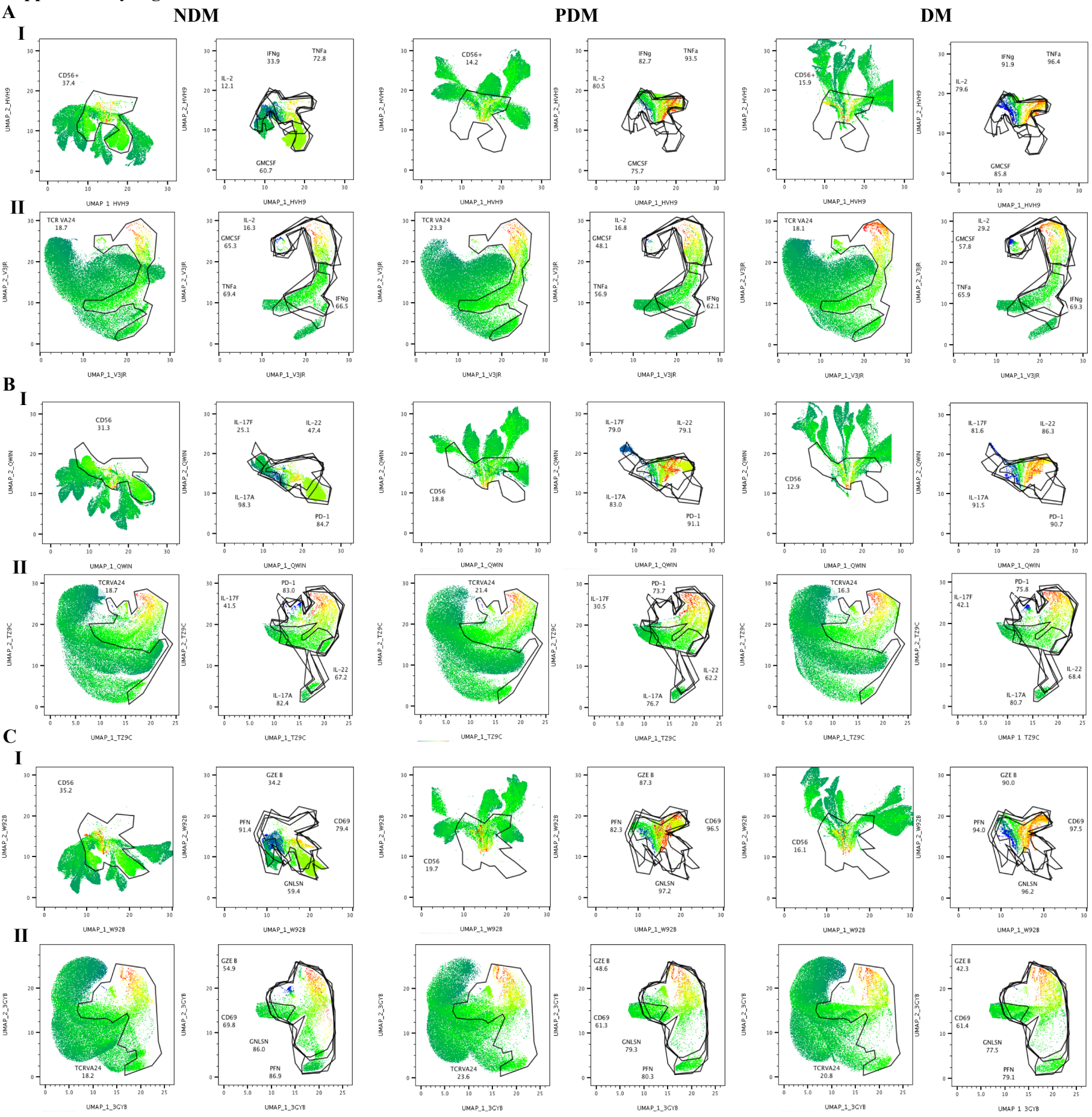

Supplement: Supplementary 1 — Figure S1: high dimensionality profile of gated NK and iNKT cells expressing Type 1/Type 17 cytokines, cytotoxic and immune markers using UMAP analysis in LTB comorbid individuals. A-C (I) PBMCs (lymphocytes) were gated for single cells (FSC-A vs FSC-H) and from which NK cells (4000-CD3−CD56+ population]) and iNKT cells (5000-CD3+ population) were downsampled. The downsampled population were concatenated and all the analysis was performed using the inbuilt plugins available in FlowJo™ 3 (Version 10). Overall gated UMAP profile of CD56+ NK cell population showing the percentage of expression from CD3− lymphocytes and further gated for Type 1 cytokine [(TNFα, IFNγ, IL-2) and GMCSF], Type 17 cytokines [(IL-17A, IL-17F, IL-22) PD-1] and cytotoxic [(PFN, GZE B, GNLSN) and CD69] markers percentage of immune clusters in LTB NDM, LTB PDM and LTB DM individuals. A-C (II) Overall gated UMAP profile of iNKT (TCRV⍺24+) cell population showing the percentage of expression from CD3+ lymphocytes and further gated for Type 1 cytokine [(TNFα, IFNγ, IL-2) and GMCSF], Type 17 cytokines [(IL-17A, IL-17F, IL-22) PD-1] and cytotoxic [(PFN, GZE B, GNLSN) and CD69] markers percentage of immune clusters in LTB NDM, LTB PDM and LTB DM individuals. [file 2422790.f1.pdf]

Th1

Th17

Cytotoxic  
Markers

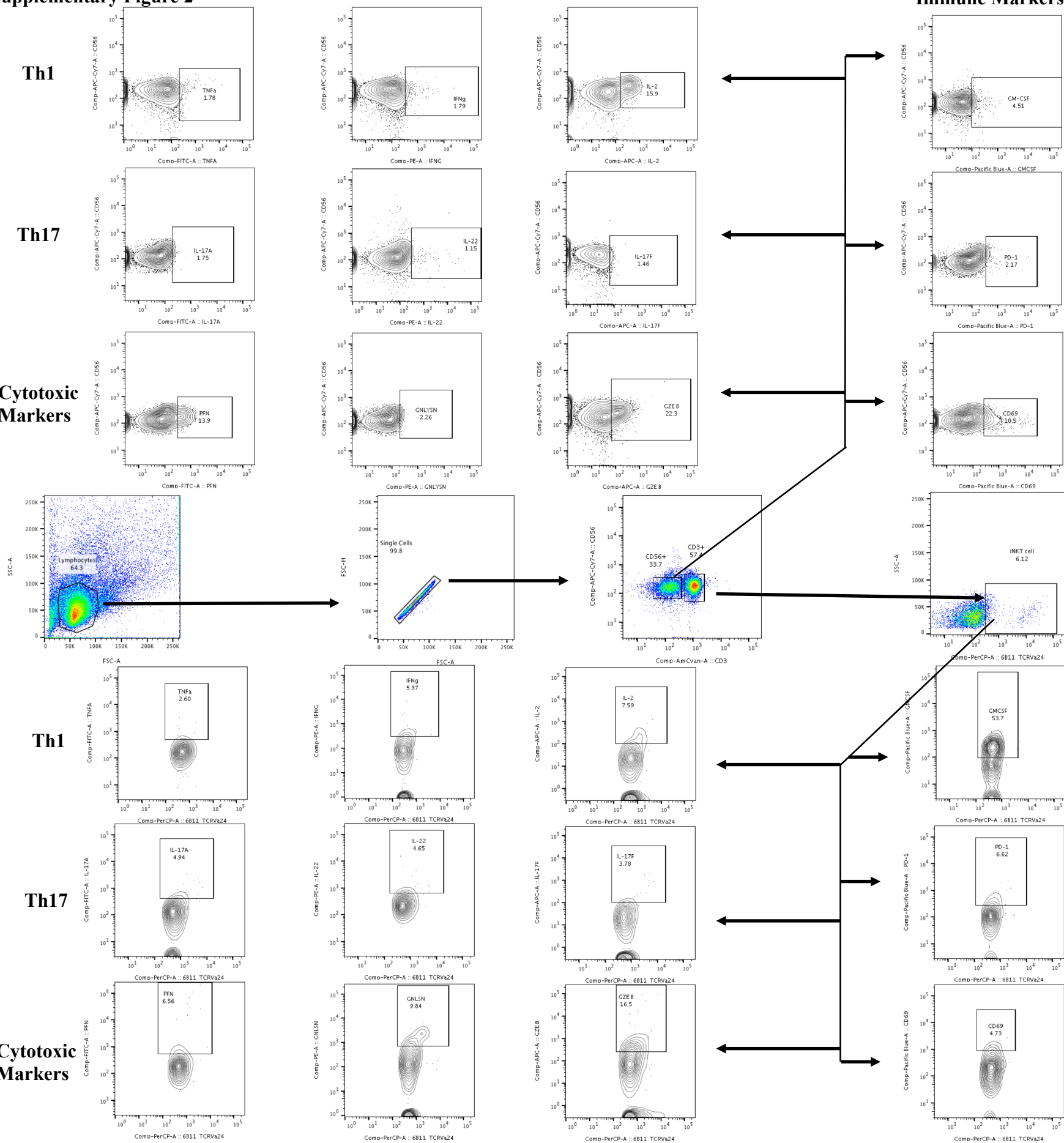

Supplement: Supplementary 2 — Figure S2: PBMCs gating strategy and representative plots for NK and iNKT cell expressing Type 1, Type 17 cytokines, cytotoxic and immune markers. Single cells were gated from lymphocytes and further gated on CD3−CD56+ population for NK cells; whereas CD3+ T cell population were selected and further iNKT cells was gated using TCRVα24 versus (vs) side scatter (SSC)-A and the frequencies of different cytokines, cytotoxic and other immune markers were indicated. [file 2422790.f2.pdf]

Supplementary Figure 3

A

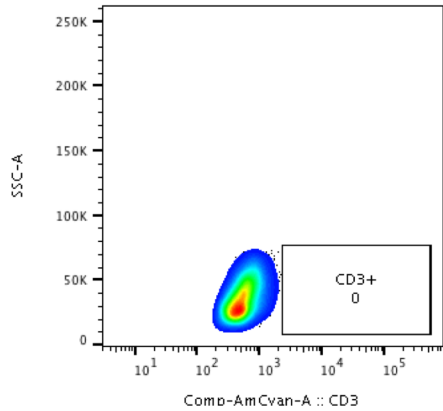

B

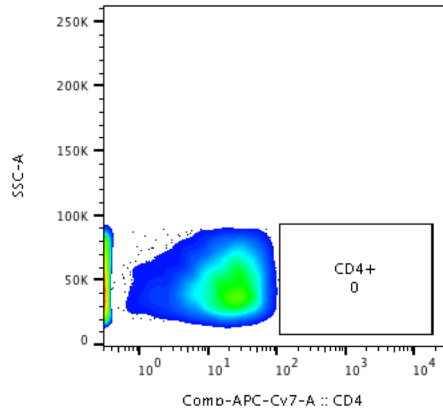

C

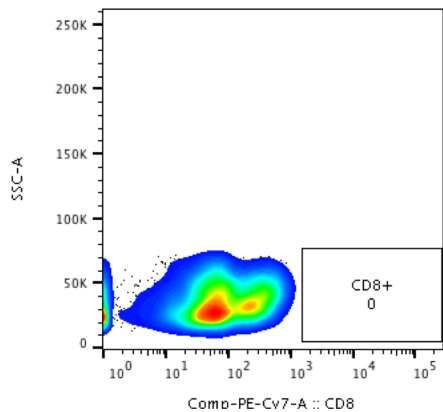

D

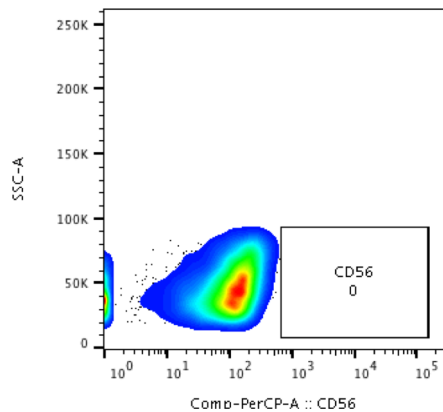

Supplement: Supplementary 3 — Figure S3: FMO activation (A. CD3, B. CD4, C. CD8 and D. CD56) markers used in the study. [file 2422790.f3.pdf]
