# Supplementary material for: High Dimensionality Reduction and Immune Phenotyping of Natural Killer and Invariant Natural Killer Cells in Latent Tuberculosis-Diabetes Comorbidity
Source: J Immunol Res. 2022 Feb 21;2022:2422790. doi: 10.1155/2022/2422790 (PMC8886750; doi:10.1155/2022/2422790)
Supplement: Supplementary 4 — Table S1: combination/panel of NK and iNKT cell antibodies used in the study. [file 2422790.f4.doc]

**Supp Table 1. NK and iNKT cell antibodies used in the study.**

| **Cell Type** | **FITC** | **PE** | **APC** | **APC Cy-7** | **PE CY-7** | **PERCP** | **AMCYAN** | **PB** |
| --- | --- | --- | --- | --- | --- | --- | --- | --- |
| NK | TNFα | IFNγ | IL-2 | CD56 | - | - | CD3 | GMCSF |
| NK | IL-17A | IL-22 | IL-17F | CD56 | - | - | CD3 | PD-1 |
| NK | Perforin | Granulysin | Granzyme B | CD56 | - | - | CD3 | CD69 |
| iNKT | TNFα | IFNγ | IL-2 | CD4 | CD8 | TCRVa24 | CD3 | GMCSF |
| iNKT | IL-17A | IL-22 | IL-17F | CD4 | CD8 | TCRVa24 | CD3 | PD-1 |
| iNKT | Perforin | Granulysin | Granzyme B | CD4 | CD8 | TCRVa24 | CD3 | CD69 |
